# Stakeholder perspectives on implementing person-centered transitional care in congenital heart disease: the STEPSTONES-Implement project

**DOI:** 10.1186/s12913-025-13645-1

**Published:** 2025-10-27

**Authors:** Markus Saarijärvi, Milena Boczar, Sandra Skogby, Carina Sparud-Lundin, Ewa-Lena Bratt

**Affiliations:** 1 Department of Cardiology, Danderyd Hospital, Stockholm, Sweden; 2 Department of Neurobiology, Care Sciences and Society, Karolinska Institutet, Stockholm, Sweden; 3 Gothenburg Centre for Person-Centred Care (GPCC), University of Gothenburg, Gothenburg, Sweden; 4 Department of Pharmacy, Uppsala University, Uppsala, Sweden; 5 Children’s Heart Center, Sahlgrenska University Hospital, Gothenburg, Region Västra Götaland Sweden; 6 Institute of Health and Care Sciences, University of Gothenburg, Gothenburg, Sweden; 7 Department of Paediatrics and Child Health, University of Cape Town, Cape Town, South Africa

**Keywords:** Adolescents, Heart defects, Congenital, Implementation science, Person-centered care, Qualitative research, Transition to adult care

## Abstract

**Background:**

Adolescents with congenital heart disease (CHD) face substantial challenges during the transition from pediatric to adult care. The STEPSTONES transition program is a person-centered, evidence-based intervention designed to support this transition, and has shown efficacy in improving patient empowerment and disease-related knowledge. However, implementing complex health interventions like the STEPSTONES transition program in real-world settings remains difficult. This study aimed to explore the barriers and facilitators to implementing the STEPSTONES transition program in Swedish pediatric cardiology clinics, using the Consolidated Framework for Implementation Research (CFIR).

**Methods:**

A qualitative study was conducted in six university hospitals in Sweden prior to implementing the STEPSTONES transition program. Semi-structured interviews were carried out with 20 stakeholders, including nurses, physicians, managers, and a patient organization representative. Data were analyzed using reflexive thematic analysis, guided by the CFIR framework, covering five domains: intervention characteristics, outer setting, inner setting, characteristics of individuals, and implementation process.

**Results:**

Facilitators to implementation of the transition program included the program’s clear structure, alignment with existing person-centered care practices, motivated local champions, and external demand for structured transitional care. Organizational readiness varied widely; clinics with managerial support, clear responsibilities, and engaged teams demonstrated higher preparedness. Barriers included limited time and resources, unclear professional roles (particularly regarding nurses’ responsibilities in delivering psychosocial components) and fragmented collaboration with adult care. Implementation efforts were often dependent on individual initiative rather than embedded in institutional routines.

**Conclusions:**

Successful implementation of the STEPSTONES transition program requires attention to multilevel contextual factors, including organizational culture, interprofessional collaboration, and leadership support. Facilitation could be a key implementation strategy to address identified barriers and enhance uptake and sustainability. These findings offer valuable insights for the broader implementation of transition programs for young people with various chronic conditions in different settings.

**Supplementary Information:**

The online version contains supplementary material available at 10.1186/s12913-025-13645-1.

## Background

Survival rates for young people with congenital heart disease (CHD) have improved significantly during the past decades, leading to a growing population of young people transitioning from pediatric to adult healthcare services [1, 2]. However, this transitional period presents critical challenges and can be marked by gaps in follow up care due to inadequate preparation for self-management leading to psychosocial challenges, limited access to specialized adult CHD services, all of which can negatively impact long-term health outcomes. Effective, coordinated transition programs are essential to address these critical challenges [3]. Both disease-specific and generic clinical guidelines, as well as patient organizations, emphasize the need for structured transition programs for adolescents with chronic conditions. Such transition programs aim to improve disease-related knowledge, self-management skills, and empowerment, thereby promoting engagement with adult care and reducing the risk of adverse health and psychosocial outcomes in young adulthood [2–5].

The STEPSTONES (Swedish Transition Effects Project Supporting Teenagers with chrONic mEdical conditionS) transition program is one of the first rigorously developed and tested interventions in Europe, specifically targeting adolescents with chronic conditions such as CHD. It is a complex intervention, consisting of a generic, person-centered, structured transition program involving three consultations with a transition coordinator (TC) over two and a half years (age 16-18.5y), covering medical, psychosocial, and lifestyle issues including independence and autonomy in young adulthood and adult care [6]. A randomized controlled trial (RCT) evaluating the efficacy of the STEPSTONES transition program for young people with CHD in Sweden, showed that the transition program was effective in improving patient empowerment (primary outcome), disease-related knowledge, transition readiness, self-image and in decreasing parental uncertainty related to the transition process (secondary outcomes) [7]. Moreover, multiple randomized controlled trials and quasi-experimental studies across various chronic conditions have demonstrated the effectiveness of transition programs in improving a range of PROM and PREM outcomes [8–11]. Despite mounting evidence and clinical promise, translating complex health interventions like transition programs into routine practice remains challenging [12]. A process evaluation embedded within the STEPSTONES-CHD trial assessed the process of implementing the program within the RCT. This evaluation found that while six out of eight program components showed high adherence, peer support components and the joint transition meeting (involving pediatric and adult providers) had lower fidelity. Factors affecting implementation included engagement from clinicians and adolescents, contextual constraints, and the absence of fully developed standard operating procedures [13–15].

Considering these insights, it is crucial to understand why and how implementation efforts succeeds or falters across different clinical settings. Implementation research frameworks such as the Consolidated Framework for Implementation Research (CFIR) offer a multilevel typology—encompassing intervention characteristics, inner and outer settings, characteristics of individuals, and the implementation process—that can guide such analysis [16]. Further, understanding determinants for implementation of evidence-based practices can aid researchers and clinicians in developing implementation strategies that can enhance implementation capacity by exploring opportunities, behaviors and motivation driving change [17]. To date, however, only a few studies have explored real-world barriers and facilitators affecting the adoption and integration of structured transition programs like STEPSTONES in pediatric cardiology units internationally [3, 18, 19]. Existing studies in various chronic conditions often highlight challenges related to staff attitudes, organizational culture, time constraints, resource limitations, and interprofessional coordination, with structural and process-related factors being largely unexplored from a multi-center perspective [20–22]. In Sweden, the STEPSTONES transition program showed encouraging results in empowerment and overall acceptance, but evidence is lacking regarding the contextual factors that enable or impede its implementation in real-life clinical settings.

Accordingly, this study sought out to identify and analyze the prerequisites to implementing the STEPSTONES transition program in six university hospitals in Sweden providing care for adolescents with CHD. Through insider perspectives we sought to uncover how organizational readiness, staff perceptions, teamwork dynamics, external demands, and implementation processes influence the adoption and sustainability of a structured transition program in real-world practice. Understanding these context-specific factors will provide crucial insights to inform future scaling of structured transition programs for adolescents with chronic conditions.

## Methods

### Aim

The aim was to explore the barriers and facilitators for implementing the STEPSTONES transition program for adolescents with CHD in pediatric outpatient cardiology clinics in six university hospitals in Sweden.

### Design

A qualitative deductive design was undertaken using the Consolidated Framework for Implementation Research (CFIR) [16]. The reporting of this study follows the Standards for Reporting Qualitative Studies (SRQR) guideline [23].

### Setting

**Fig. 1:**
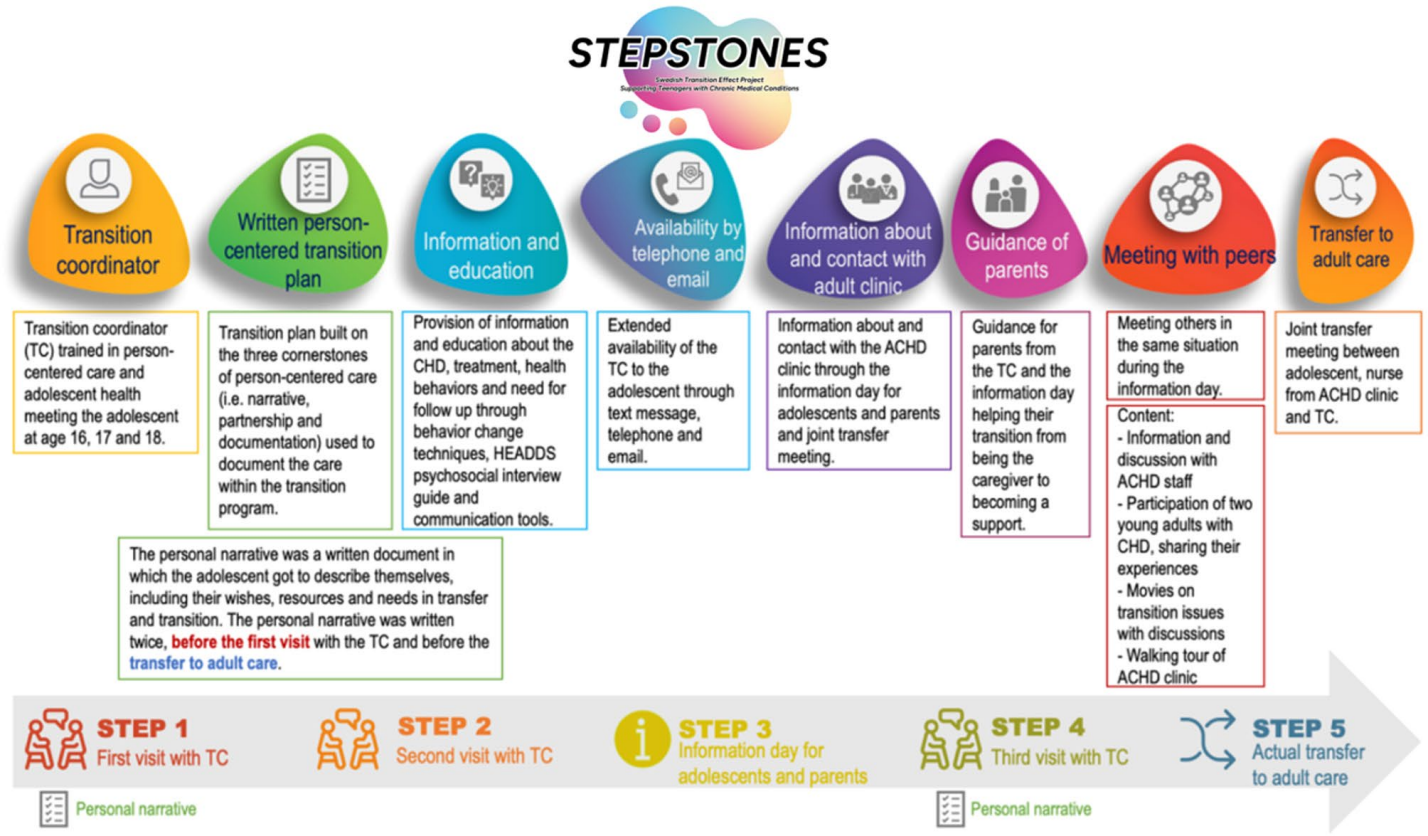
Outline of the STEPSTONES transition program. Source: reproduced with permission from BioMed Central Ltd [13]

In Sweden, care for people with CHD is centralized to the university and regional hospitals where follow-up is determined based on anatomical complexity of the CHD. Medical follow-up ranges from yearly check-ups to every three-five year according to international guidelines [4]. During 2017–2021 a RCT was conducted evaluating the efficacy of a person-centered transition program (STEPSTONES transition program) for adolescents with CHD in transition to adulthood and adult care (age 16–18.5y) [24]. In short, the transition program consists of eight components implemented in five steps (Fig. 1). The RCT concluded that the transition program was effective in improving patient empowerment (primary outcome) [7]. Following this, we established the Stepstones-Implement project with the aim to implement the Stepstones transition program in Swedish healthcare, using pediatric cardiology as a sample case for a broader future implementation. In 2022, we approached all seven university hospitals in Sweden that provide care for adolescents with CHD to implement the Stepstones program; six of these hospitals joined the Stepstones-Implement project. Consequently, all included hospitals were invited to participate in this preparatory study before implementation of the program.

### Participants

To achieve the objective of the study, we invited healthcare professionals working at the six pediatric outpatient clinics participating in the Stepstones-Implement project to semi-structured interviews before the implementation of the transition program. Inclusion criteria were: (1) working as a registered nurse, physician or manager at an outpatient clinic providing care for adolescents with CHD, (2) having at least 2 years of working experience from the outpatient clinic, as we wanted the participants to have lived experience of providing transitional care for young people with CHD. No formal exclusion criteria were stated. We used a purposive sampling strategy where we strived for maximum variation in professional roles (i.e., nurse, physician or manger), age, sex and professional experience [25]. Twenty-five individuals were identified working at the six participating clinics, with five individuals declining or being unreachable, leading to a final sample of 20 participants. To achieve a broader societal perspective on barriers and facilitators we also performed an interview with the chair of The Swedish Heartchild Foundation.

### Data collection

Twenty semi-structured interviews were carried out between 2022 and 2023 over the telephone or via Microsoft Teams™ according to the participants preferences [26]. The interview guide was based on the five domains of the CFIR framework i.e., *Intervention characteristics, Outer setting, Inner setting, Characteristics of individuals*, and *Implementation process* [16] (Supplemental file 1). Interviews lasted between 30 and 52 min, were audio recorded using an external recording device, and transcribed verbatim by a research assistant. The interviews were carried out by the first author (MS) who is a RN and researcher in transitional care. The interviewer had no professional working relationship with the interviewees at the time of the interview and had experience of conducting qualitative interviews on the research topic [13, 15].

### Data analysis

A deductive reflexive thematic analysis according to Braun and Clarke [27] was performed using the CFIR as deductive framework [16]. Table 1 provides a description of the CFIR that guided the coding process and analysis. All analyses were performed in Nvivo version 14 by two of the authors (MS and MB). The analysis started by the both authors reading the interview transcripts several times to get a grasp of the whole. We then identified meaning units relevant to the aim of the study and categorized them into one of the five CFIR domains, guided by the framework’s descriptions (Table 1). The meaning units were then merged to form codes that answered to the aim of the study. Finally, these codes were then narratively summarized within each domain of the CFIR with quotes to illustrate and ensure transparency of the analysis. The two authors responsible for the analysis (MS and MB) met regularly during the analytic process to compare and critically discuss emerging findings, enhancing the trustworthiness of the results. Finally, all co-authors read, revised and discussed the results critically to ensure trustworthiness of the analysis [28]. The analysis were performed entirely in Swedish and were translated to English using a Large-Language-Model when writing the results of the manuscript.

**Table 1:** CFIR domains with descriptions [16]

Domain	Description
Intervention Characteristics	Refers to the features of the intervention itself that may influence implementation. This includes aspects such as the source of the intervention, its perceived evidence strength and quality, relative advantage compared to current practice, adaptability to local context, trialability, complexity, design quality, and associated cost.
Outer Setting	Encompasses the external influences on implementation, including the broader economic, political, and social context. This domain covers patient needs and resources, external policies and incentives, peer pressure or competition.
Inner Setting	Focuses on the internal context of the implementing organization. It includes structural characteristics, networks and communication, culture, implementation climat, and readiness for implementation (leadership engagement, available resources, access to knowledge and information).
Characteristics of Individuals Involved	Addresses the attributes of the people involved in implementation, such as their knowledge and beliefs about the intervention, self-efficacy, individual stage of change, identification with the organization, and other personal attributes that influence behavior and adoption.
Implementation Process	Refers to the active, purposeful steps taken to implement the intervention. Key elements include planning, engaging relevant stakeholders (e.g., opinion leaders, champions, external change agents), executing the implementation, and reflecting and evaluating to inform continuous improvement.

## Results

Demographic characteristics of participants are described in Table 2. The findings are thematically organized according to the five CFIR domains [16]: *Intervention characteristics, Outer setting, Inner setting, Characteristics of individuals*, and *Implementation process* as well as a detailed description of the related barriers and facilitators according to the aim of the study (Table 3).

**Table 2:** Demographic characteristics of participants

	Nurses (*n* = 9)	Physicians (*n* = 4)	Managers/patient organization chair (*n* = 7)
Years of professional experience*	15 (2–30)	14.75 (5–20)	5.3 (2–13)
Sex **			
Female	9	3	7
Male	0	1	0
Center 1	2	-	1
Center 2	2	1	1
Center 3	2	-	1
Center 4	1	1	2
Center 5	1	1	1
Center 6	1	1	1

*Mean and range **absolute number

**Table 3:** Barriers and facilitators to implementation of the STEPSTONES transition program according to the consolidated framework for implementation research (CFIR) [16]

CFIR Domain	Facilitators	Barriers
Intervention characteristics	• Clear structure • Useful tools such as HEADDS, transition plan, active learning • Good applicability and alignment to current practices	• Scope of program • Insecurity on the level of adaptation and flexibility of the program
Outer setting	• External demands from patients, caregivers and society on the need for structured transitional care	• Unequal care • Limited support and care to vulnerable groups, i.e., neurodevelopmental conditions • High expectations on implementation but difficulties in finding structures
Inner setting	• Engaged local teams • Existing usual care practices for transitional care that the transition program can dock in too	• Lack of resources, e.g., time, staffing, structures • High staff turnover • Unclear responsibilities within the team
Characteristics of the individuals involved in implementation	• Strong engagement in the team • Local implementation champions • High professional motivation in providing excellent care	• Uncertainties regarding nurse’s role and responsibility in transitional care • Lack of self-efficacy in performing all components of the program, for instance sexual and mental health conversations
Implementation processes	• Local drive to facilitate implementation • Positive attitudes from managers • Good examples of positive implementation initiatives	• Lack of formal coordinator role in some settings • Lack of clear evaluation or follow-up from management • Implementation is dependent on champions

### Intervention characteristics – perceived features of the transition program

The STEPSTONES transition program was perceived as a well-designed, evidence-based, and structured program. Most interviewees described the program positively and felt it provided clear tools to support adolescents in their transition to adult care. Components such as the HEADDS (Home Education Activities Depression Drugs Sexuality) psychosocial interview guide, person-centered transition plan, and individualized and person-centered education and learning were seen as concrete and applicable in practice and facilitators for implementation.

*These were good and concrete tools, and there was nothing that contradicted how we previously worked. It felt like a good next step. - Interview 10, Manager*

Several respondents noted that STEPSTONES largely aligned with their current working methods, especially where youth-adapted approaches were already in place. This suggests that the program was viewed as compatible with existing practices and a facilitator for implementation. At the same time, there were perceived barriers, such as concerns about the program’s scope and how realistic it is to implement all components in everyday clinical settings with limited resources. Another issue raised was the flexibility of the program, specifically how much its components could be adapted to local contexts without compromising its core structure.

*Looking at the program as a whole, it’s always a matter of resources—being able to get through all the steps. For example, the idea of sitting down together with the adult clinic… I’m not sure we’ll be able to make that happen. Partly because we’re short on space, and sometimes it’s hard even to get the adolescent to come. And we don’t have that many staff at our clinic right now, and not that many who are experienced, so that might be a concern, you could say. – Interview 14, Manager*

### Outer setting – external needs, demands, and relationships with adult care

External factors were seen as having a significant influence on implementation. Many interviewees describe pressure from patients, families, and societal actors such as patient organizations to improve the transition between pediatric and adult care for adolescents with CHD. There was a recurring perception that adolescents’ needs were not adequately addressed, especially among those with milder heart defects.

*For those without complex heart defects, sometimes only a referral is sent to adult care – not everyone receives a structured conversation beforehand.” - Interview 20, Physician*

One of the most prominent barriers in the interviews concerned the variation and inconsistency in collaboration between pediatric and adult cardiology. While some clinics had established routines and close working relationships with adult services, others described a fragmented transition process that often relied on individual initiative rather than formalized structures. Further, a recurring challenge was the lack of coordination and clear accountability on the adult care side. Several interviewees expressed concern that no one was assigned overall responsibility for ensuring continuity, in contrast to pediatric teams, which were more often viewed as attuned to adolescents’ psychosocial needs and the importance of a structured and supportive transition.

*The main problem is that there’s no one on the adult side who ties everything together. Everyone says, ‘we’re doing our part,’ but no one sees the whole picture. – Interview 16, Physician*

Logistical and structural barriers further complicate collaboration. Respondents described difficulties in aligning schedules between teams and maintaining contact over time:

*It might be that I feel this way because in recent years we’ve completely lost contact with them [adult care]. It’s always difficult when there are many parties involved, families, us, adult care, maybe a social worker. As soon as it involves multiple people, it becomes hard to coordinate. – Interview 16, Physician*

Patient organizations also expressed concerns that adolescents may be left without support, underscoring the need for a formalized transition program. Additional external pressure stemmed from national guidelines and initiatives aimed at integrated care, further legitimizing implementation. Respondents also emphasize that the transition is particularly vulnerable for youth with neurodevelopmental conditions or lower socioeconomic status, indicating that a structured transition program like STEPSTONES could mitigate health inequities.

*Some children have disabilities, some don’t – they just have a heart defect, and maybe they don’t need help with that part. But there are others who have a much harder time with the transition, because they might also have ADHD, for example. And then we might need to step in more. So, I think you really need to be able to sense the situation and adapt yourself in this transitional work. – Interview 9, Nurse*

### Inner setting – the impact of organizational conditions and culture

The organizational context was perceived as playing a decisive role in implementation of the STEPSTONES transition program. Some clinics benefitted from strong managerial support, established routines for transfer and transition, and a culture that supported person-centered care. In these settings, implementation conditions were considered as favorable and facilitating. Several respondents reported they had already conducted youth visits, joint meetings with adult care, or HEADDS interviews before the implementation of STEPSTONES and that the program was seen as a complement.

*Here, we start at age 12 by offering youth the opportunity to meet with us alone while their parents wait outside. - Interview 9, Nurse*

Several nurses expressed that the STEPSTONES structure resonated with their professional values. Tools such as HEADDS and person-centered conversations provided tangible methods to address complex issues like independence and sexuality. In addition, at certain sites, the nurses received clear support from leadership, which included dedicated time and resources.

*My main task is to make sure that the transition coordinator gets the time, space, structure, and expectations to do this work, and that she feels supported. – Interview 11, Manager*

Several organizational barriers within the inner setting of pediatric cardiology were identified as critical challenges to the implementation of the STEPSTONES program. Although nurses play a central role in the STEPSTONES model, several interviewees noted that their actual duties in daily clinical work were often restricted to administrative tasks or basic clinical measurements. This limited their capacity to act as true care coordinators or to engage meaningfully in transitional counseling with adolescents. In some settings, the specialized competencies of trained nurses were underutilized, which hindered the program’s effectiveness.

*The specialist nurse’s role is often reduced to measurements and admin, rather than active coordination. – Interview 18, Physician*

The absence of formalized routines or infrastructure for transitional care was frequently mentioned as a barrier. Several participants expressed that implementation success largely depended on the motivation and knowledge of individual staff members rather than systemic support. As a result, the process for transition could vary greatly between teams, even within the same clinic.

*It’s really up to the individual person responsible in each team. People value different things, and without clear guidance, you just do what you think is best. – Interview 6, Nurse*

In many clinics, care teams were viewed as operating in silos, with limited communication or knowledge-sharing about how others approach transition. This fragmented structure restricted opportunities for standardization and collective learning. Even promising practices developed by one team often remain isolated and unshared.

*Teams work quite individually here, and we usually don’t know what other teams are doing. We’ve started having meetings where each team presents something good that they’re doing, but that’s fairly recent. – Interview 12, Manager*

Another barrier relates to the position of middle managers or transition coordinators who are responsible for leading the implementation but lack formal authority to allocate resources or staff. These individuals often find themselves needing to lobby for support within a rigid system, which could delay or complicate implementation efforts.

*I can’t say, ‘we’ll assign a nurse and free up time.’ That’s not within my authority. I can present the case to senior management, but it depends on whether they choose to prioritize it. – Interview 11, Manager*

### Characteristics of individuals involved – staff knowledge, attitudes, and engagement in transitional care

Individual-level factors were evident in the interviews acting both as barriers and facilitators. Many nurses and some physicians showed strong personal commitment to adolescents’ health and development. Several had spearheaded new approaches even in the absence of formal structures. It was clear that local champions had been crucial in raising awareness about the need for structured transition.

*She is truly a champion – which is the key to success in this area. She drives the work and has a thoughtful approach. - Interview 19, Physician*

However, there were some resistances or areas for uncertainty in the implementation. Some physicians raised concerns about nurses leading conversations on sensitive topics such as sexuality or mental health, which could be considered as threatening the transition program’s internal acceptance. At the same time, some nurses reported feeling unsure of their authority to take on a more active role, despite receiving training.

*In our clinic, some physicians don’t think nurses should talk about sexuality and things like that. And then it becomes a bit difficult, because even if you’ve completed the training, you’re not sure if you’re really allowed to take on that role.— Interview 19, Physician*

### Implementation process – planning, anchoring, and implementation

Perceptions on how the transition program was going to be implemented varied widely between clinics. Where formal anchoring of the implementation in the staff group and managerial level were present, good examples of integration into daily routines were already noted. In other settings, the absence of a clear strategy for implementation meant that efforts would rely on individual initiatives, presenting a clear barrier.

*We had a nurse who was really passionate about the transition process… She worked closely with our ward doctor to determine when it was time [to initiate transition] and tried to develop packages for different key ages. But when we were told not to proceed further at that point, we had to back off. Later, there were reorganizations, and that nurse left. No one else picked up the responsibility, so we’ve kept a lower profile since then.— Interview 13, Manager*

The participants called for clearer support from leadership, established cross-level collaboration routines between pediatric and adult care, and opportunities for follow-up and reflection of the implementation efforts. Some emphasized the need for a designated transition coordinator or permanent function responsible for maintaining continuity in the program.

## Discussion

This study aimed to identify barriers and facilitators to implementing the STEPSTONES transition program for adolescents with CHD across six university hospitals in Sweden. Using the CFIR as a guiding framework, we found that prerequisites for successful implementation was influenced by a complex interplay between intervention characteristics, organizational context, individual staff attributes, and implementation processes. This findings echo previous studies investigating the experiences and outcomes of implementing transitional care interventions in different settings showing that success factors have been local adaptation and tailoring to overcome implementation challenges [29], having local champions and leadership support [30] as well as the impact of organizational behaviours and context [20]. To the best of our knowledge, this is the first study to investigate barriers and facilitators to the implementation of transition programs using the CFIR. This contributes to the broader field of implementation science, as well as providing a solid theoretical foundation for understanding these challenges within the context of transitional care.

Our results demonstrate that the STEPSTONES transition program was perceived as highly structured, evidence-based, and well-aligned with the ethos of person-centered care. These features are known facilitators for implementation and echo findings from previous research on structured transition programs in various chronic conditions, where perceived coherence and adaptability of the intervention are critical for uptake [11, 31]. However, participants in this study also expressed concerns about the program’s scope and flexibility, especially in settings with constrained resources. This reflects broader challenges in translating complex interventions into practice, particularly when the core components are perceived as rigid or misaligned with local capacity [12].

The outer setting, including societal and patient demands, also emerged as a driver of implementation. Stakeholders emphasized widespread recognition of the need for structured transitional care, especially for vulnerable populations such as those with neurodevelopmental conditions—consistent with global calls for equity in transitional care [3]. Nonetheless, fragmented communication between pediatric and adult care providers and the lack of formalized joint responsibilities mirrored international findings [18, 19], highlighting the systemic nature of these barriers and that future implementation efforts should target not only pediatric care but also adult care.

Organizational readiness varied considerably across clinics. Where leadership engagement and structural support existed, prerequisites for implementation were considered more robust. In contrast, sites with unclear responsibilities, high staff turnover, or resource limitations were more uncertain about how to integrate the program into routine care. These observations underscore the importance of organizational culture and support mechanisms in the implementation of complex interventions [20].

An important insight from this study was the crucial role of local champions in driving implementation. Indeed, local clinician champions have proven to be pivotal in achieving successful implementation of evidence-based practices [32]. Nevertheless, while local champions are important for initiating behavior change, they alone are insufficient to maintain such change over time [33]. In our context, nurses were identified as key facilitators—yet they also faced uncertainty regarding role boundaries and acceptance by physicians, particularly in addressing sensitive topics such as sexual and reproductive health. These findings align with previous studies indicating that professional hierarchies and unclear role definitions can hinder implementation [21]. It was noted that there were different perceptions of the nurse’s role in the transition program depending on clinic, specifically from some physicians. Highlighting the importance of context-specific implementation strategies by strengthening interprofessional collaboration and clarifying roles, which may enhance confidence and uptake among staff [17].

A caveat for the implementation was that many participants considered previous implementation efforts often dependent on individual initiative rather than being embedded within a systematic process. Here, clinics with well-anchored implementation strategies, designated time for nurses acting as transition coordinators, and follow-up monitoring might result in a better future integration of the STEPSTONES transition program at these clinics. Conversely, reliance on individual staff members without systemic support might lead to variability and risk of program discontinuation. This confirms the proposition that successful implementation requires both individual motivation and institutional anchoring [16, 17].

Our findings indicate that based on the need for local adaptations, altering and understanding context and promoting the use of clinician champions, one promising implementation strategy to mitigate these barriers and strengthen facilitators could be facilitation. This strategy aims at supporting individuals, teams and organizations in applying evidence-based practices within clinical settings. Core functions being enabling individuals and teams to analyze and improve practice, capacity building, and creating a supportive context by aligning the team efforts with leadership support [34]. Facilitation has shown to be effective in increasing both uptake and maintenance of evidence-based practices [35]. Based on our findings, this strategy could balance the need for both initating and mainting behavior change [33] as well as fostering a sense of ownership of the innovation in clinical practice.

### Methodological considerations

A strength of this study is the inclusion of multiple stakeholder perspectives across six different clinical contexts, enhancing the transferability of findings. The use of the CFIR facilitated systematic analysis across multiple levels of implementation. However, the study is limited to the pre-implementation phase, is cross-sectional in its’ design, and does not capture how barriers and facilitators evolve over time which is a important consideration in the process of implementing evidence-based knowledge into practice [36]. Further, as we sampled only three to four HCPs from each participating outpatient clinic we analysed the interview transcripts as a whole, while striving for identifying variations and common themes in experiences of barriers and facilitators to implementation [37]. As such, this made it difficiult to perform a cross-case analysis that would have provided more depth and breadth to the results. To manage these limitations and explore how implementation success over time, we are planning an extensive process evaluation of the STEPSTONES-Implement project to understand implementation outcomes as well as the mechanisms leading to successful or unsuccessful implementation [38]. Moreover, while participants represented different professional roles, the small number of physicians limits generalizability across medical staff. Furthermore, the marked gender imbalance, only one male participant, may have introduced bias or influenced the study outcome.

## Conclusions

This study highlights the complex, multilevel nature of implementing a structured, person-centered transition program for adolescents with chronic conditions. Using the CFIR, we identified a range of facilitators—such as perceived program coherence, staff engagement, and alignment with person-centered values—as well as key barriers, including resource limitations, unclear role definitions, and variability in organizational readiness. The findings underscore the importance of context-sensitive implementation strategies that not only address individual motivation and team dynamics but also institutional structures and leadership engagement. Importantly, the implementation strategy of facilitation could be a promising approach to enhance uptake by supporting team capacity, aligning efforts across levels of the organization, and embedding the intervention into routine practice. Future implementation efforts within transitional care should adopt a systematic, coordinated approach that includes designated transition coordinators, clear interprofessional collaboration pathways, and continuous leadership support to ensure program sustainability and equity in care. These insights can inform the broader implementation of transition programs in pediatric cardiology and other health care contexts.

## Supplementary Information

Below is the link to the electronic supplementary material.

Supplementary Material 1

## Data Availability

The datasets analysed during the current study are available from the corresponding author on reasonable request.

## Abbreviations

CFIR: Consolidated Framework for Implementation Research
CHD: Congenital Heart Disease
HEADDS: Home Education Activities Depression Drugs Sexuality
RCT: Randomized Controlled Trial
SRQR: Standards for Reporting Qualitative Studies Guideline
STEPSTONES: Swedish Transition Effects Project Supporting Teenagers with Chronic Medical Conditions
TC: Transition Coordinator
