# Supplementary material for: Stakeholder perspectives on implementing person-centered transitional care in congenital heart disease: the STEPSTONES-Implement project
Source: BMC Health Serv Res. 2025 Oct 27;25:1414. doi: 10.1186/s12913-025-13645-1 (PMC12560310; doi:10.1186/s12913-025-13645-1)
Supplement: Supplementary file 1 — Supplementary Material 1 [file 12913_2025_13645_MOESM1_ESM.docx]

**Additional file 1**

**Overall aim:** To describe healthcare professionals' and managers’ perceptions of the facilitators and barriers for implementing the STEPSTONES transition program for young people with congenital heart disease.

**Interview Guide – Interviews with Healthcare Professionals**

1. Can you describe how care is organized at your unit for adolescents transitioning to adult healthcare?
2. (Description of the components of the transition program.) Based on this description, what are your thoughts on the transition program?
3. Which parts of the program do you think will be the most difficult to implement?
4. In what ways would the program need to be adapted to meet your needs?
5. As a nurse/physician, how do you view your role in the program?
6. As a nurse/physician, how do you perceive the collaboration with other professionals within the framework of the transition program?
7. What factors in your workplace do you think promote the implementation of the transition program?
8. What positive benefits could result from implementing the program at your unit?
9. What organizational factors (factors outside the workplace) do you think influence the implementation of the program?
10. What kind of support would you need when implementing the program?
11. If there were a person to support and coach the implementation of the program, who should this person be and what qualities should they have?
12. That was all the questions I had. Is there anything you would like to add?

**Interview Guide – Interviews with Managers and Patient organization representative**

1. Can you describe how care is organized at your unit for adolescents transitioning to adult healthcare?
2. (Description of the components of the transition program.) Based on this description, what potential barriers do you see to implementing the transition program at your unit?
3. Which parts of the program do you think will be the most difficult to implement?
4. As a manager, how do you view your role in implementing the program?
5. What factors in your workplace do you think promote the implementation of the transition program?
6. How do you perceive the readiness for change in your organization?
7. What organizational factors (outside the workplace) do you think influence the implementation of the program?
8. What kind of support would your unit need when implementing the program?
9. If there were a person to support and coach the implementation of the program, who should this person be and what qualities should they have?
10. That was all the questions I had. Is there anything you would like to add?
